# Some Clouds Have a Silver Lining: Paradoxes of Anthropogenic Perturbations from Study Cases on Long-Lived Social Birds

**DOI:** 10.1371/journal.pone.0042753

**Published:** 2012-08-22

**Authors:** Daniel Oro, Juan Jiménez, Antoni Curcó

**Affiliations:** 1 Institut Mediterrani d'Estudis Avançats IMEDEA, CSIC-UIB, Esporles, Spain; 2 Conselleria de Medio Ambiente, Generalitat Valenciana, Valencia, Spain; 3 Parc Natural del Delta de l'Ebre, Deltebre, Spain; Monash University, Australia

## Abstract

In recent centuries and above all over the last few decades, human activities have generated perturbations (from mild to very severe or catastrophes) that, when added to those of natural origin, constitute a global threat to biodiversity. Predicting the effects of anthropogenic perturbations on species and communities is a great scientific challenge given the complexity of ecosystems and the need for detailed population data from both before and after the perturbations. Here we present three cases of well-documented anthropogenic severe perturbations (different forms of habitat loss and deterioration influencing fertility and survival) that have affected three species of birds (a raptor, a scavenger and a waterbird) for which we possess long-term population time series. We tested whether the perturbations caused serious population decline or whether the study species were resilient, that is, its population dynamics were relatively unaffected. Two of the species did decline, although to a relatively small extent with no shift to a state of lower population numbers. Subsequently, these populations recovered rapidly and numbers reached similar levels to before the perturbations. Strikingly, in the third species a strong breakpoint took place towards greater population sizes, probably due to the colonization of new areas by recruits that were queuing at the destroyed habitat. Even though it is difficult to draw patterns of resilience from only three cases, the study species were all long-lived, social species with excellent dispersal and colonization abilities, capable of skipping reproduction and undergoing a phase of significant long-term population increase. The search for such patterns is crucial for optimizing the limited resources allocated to conservation and for predicting the future impact of planned anthropogenic activities on ecosystems.

## Introduction

Ecological disturbances or perturbations affect ecosystems because they are liable to have severe effects on the physical environment and its biological components. When perturbations are very severe and cause profound effects on ecosystems they are defined as catastrophes, which are outliers of environmental stochasticity. Catastrophes are difficult to predict and as such the monitoring of their ecological consequences at species, community and ecosystem levels represents a real challenge [1]–[3]. Natural perturbations, whether geological (e.g. volcanic eruptions, floods and earthquakes) or biological (e.g. the appearance of alien species, infestations by parasites or toxic events), are common at both ecological and evolutionary scales and can trigger sharp shifts in ecosystems via changes in dynamic equilibrium states (i.e. regime shifts) and extinctions [4]. This is especially true when the consequences are global and change most ecosystems, for instance following a giant meteor strike.

In recent centuries, and above all over the last few decades, human activities have generated anthropogenic perturbations and catastrophes that, when added to natural perturbations, constitute a global threat to biodiversity [5]–[6]. One of the main characteristics of perturbations of human origin is their capacity to affect the vital rates that are more buffered against environmental variability by natural selection, which translates into a highly negative influence on the annual multiplication rate of a population. For instance, large adult elephant males have been hunted for their ivory for several thousand years; being long-lived organisms, the vital rate that is proportionally best buffered against ‘natural’ environmental variability is adult survival [7]. The killing of adult elephants has meant that their populations have declined dramatically and still have to recover [8]. Species-specific variability in resilience probably depends on a species' position in the trophic web [9]. Vertebrates have the slowest and poorest resilience [10] and the population dynamics of top predators are most affected by perturbations [11]. The organisms evolving in habitats that historically have been most affected by natural perturbations (e.g. flooding or wildfires) and that are dependent on new habitats appearing after perturbations may eventually turn out to be more resilient to anthropogenic perturbations [1], [12].

Further characteristic threats of anthropogenic perturbations and catastrophes are associated with the rate of change (faster than many natural perturbations [13], but see [14]); those human perturbations are also characterised by being multifactorial, widespread and novel. Thus, aside from the interest in studying the features of natural perturbations (i.e. their frequency, severity and predictability) and the responses of populations and communities to such events [15]–[17], increasing concern is being shown for the assessment of disturbances of human origin, especially those considered as catastrophes [5], [18]–[19]. The nuclear accident in Chernobyl (1986), the Exxon Valdez oil spill in Alaska (1989) and the heavy metal toxic spill in Doñana (1998) are all outstanding examples [20]–[22]. Some less severe but much more frequent perturbations include habitat loss and fragmentation (e.g. from logging and the construction of infrastructures), direct mortality (such as fisheries bycatch and poaching) and continued pollution [23].

Here, we present three cases of well-documented anthropogenic perturbations affecting three species of birds: Montagu's harriers (*Circus pygargus*), griffon vultures (*Gyps fulvus*) and greater flamingos (*Phoenicopterus roseus*). We assess the impact of those disturbances and the response and resilience of the bird populations involved. The study cases in common involve long-lived, slow reproducing avian social species [24]–[26] and our analysis and conclusions should thus be considered with caution and applicable to species with similar life history characteristics. The perturbations consisted of different forms of habitat loss and deterioration, one of the main threats to contemporary ecosystems: the building of an airport that destroyed the core breeding and foraging habitat of Montagu's harriers, the construction of a windfarm that led to severe bird mortality and a decrease in fertility due to fatal collisions of griffon vultures (*Gyps fulvus*), and the construction of a dyke, which reduced the isolation of the breeding site and its protection from terrestrial predators for a colony of greater flamingos. We expected population sizes to be negatively affected by the perturbations in question, especially when they affected not only fertility but also adult survival probability. We selected cases for which we had data generated by detailed monitoring programs from before and after the perturbations, and for both the study species, the ecosystem and its characteristics. This allowed us to interpret with precision the consequences of the perturbation at population level and identify the demographic mechanisms at work. These three species are all long-lived and so adult survival is the parameter with highest demographic sensitivity in their population dynamics; flamingos and vultures have generation times of ca. 22 years, whereas harriers have a shorter one (ca. 15 years). Thus, we expected a greater negative effect of perturbations for vultures than for harriers and flamingos because, of the three, the former is the only species for which both fertility and adult survival was affected [25].

## Methods

We searched for well-documented events of anthropogenic perturbations affecting species for which information on population sizes was available before and after these events took place. We were not interested in the biological responses affecting other parameters (e.g. behavioural, reproductive or physiological parameters) and studied only an integrative parameter of population fitness, that is, the population growth rate. We chose not to search the literature due to the bias caused by the partial publication of results (i.e. the hiding or non-publication of negative results) [27]. As a by-product of ongoing monitoring schemes we were involved in, we had access to three individual case studies. We also searched for species that had been monitored in Spain for a number of years and for documented human perturbations affecting whole local populations at a precise moment in time. We discarded those cases because missing values in many of the time series of population sizes in other potential species precluded a thorough analysis.

**Figure 1:**
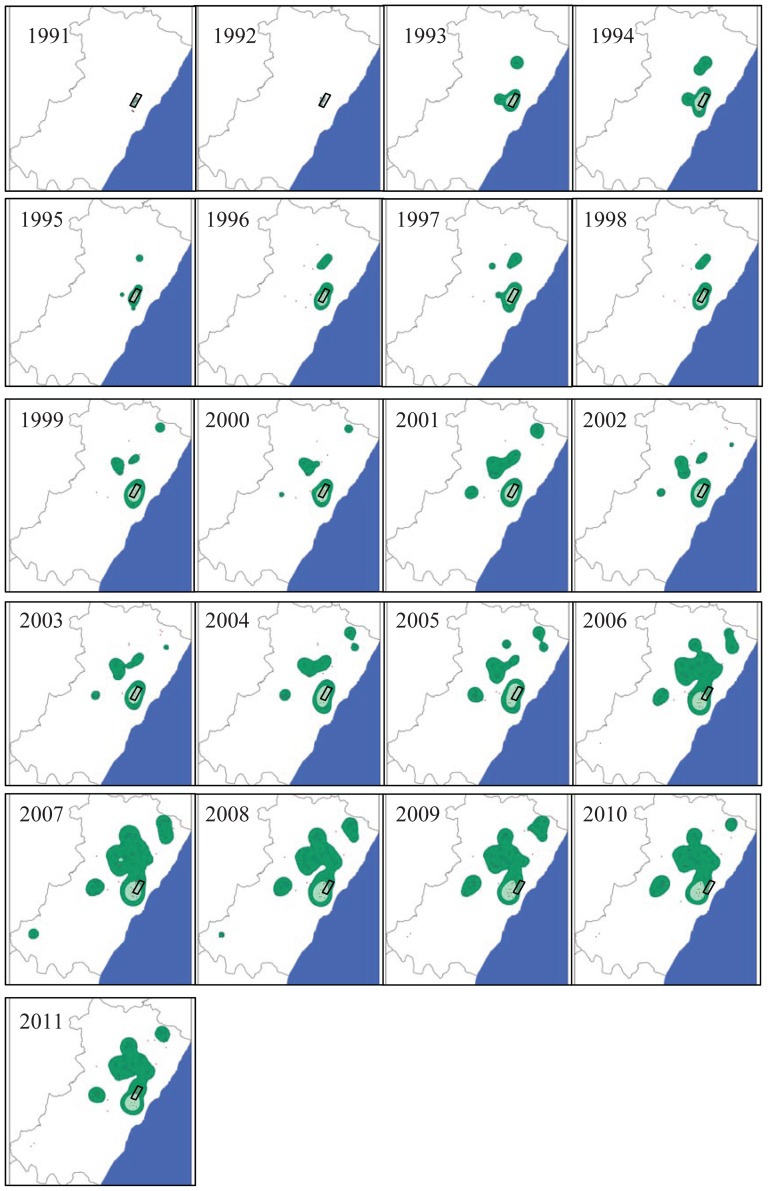
Spatial colonization of Montagu's Harriers at the study area. Breeding range of the area occupied by Montagu's harriers in the study area (Castellon province, its limits shown by black lines, ca. 6600 km^2^) by using a spatial probabilistic Kernel analysis (with 50 and 90% probabilities showed by green and shaded blue areas respectively) for the period 1991–2011 using the nests (showed by black dots) localized in a UTM 1×1 km square. The black parallelogram shows the area occupied by the airport; we note here that the airport was built in 2005. The blue part corresponds to the Mediterranean Sea.

The three case studies, all from north-east Spain, had been well-monitored by our research teams before and after an anthropogenic perturbation. Montagu's harriers are semi-colonial ground-nesting raptors. The study population inhabits a natural Mediterranean (not cereal crops) habitat, dominated by sclerophyllous Mediterranean shrublands of *Querco cocciferae–Pistacietum lentisci* located between 10 and 30 km from the coast [28]. Field searches were conducted between April and July each year to locate nests and estimate the breeding population size. Fieldwork effort was high (covering all the suitable potential habitats) and similar over the years so we assumed that undetected nests were few and did not bias the trend in population size. The Minimum Convex Polygon occupied by nesting harriers increased from ca. 6 km^2^ in the early 1990s to ca. 2,190 km^2^ in 2011 (see below). The breeding population time-series covers 25 years (from 1987 to 2011). The airport was built in 2005 and overlapped spatially with the historical core habitat of the harrier population (Fig. 1); in that year, ca. 10% of the population was directly affected because the spatial spread of the population was initiated long before during the mid 1990's (see Results). All the nests inhabiting the area where the airport was built disappeared because the habitat was totally destroyed.

**Figure 2:**
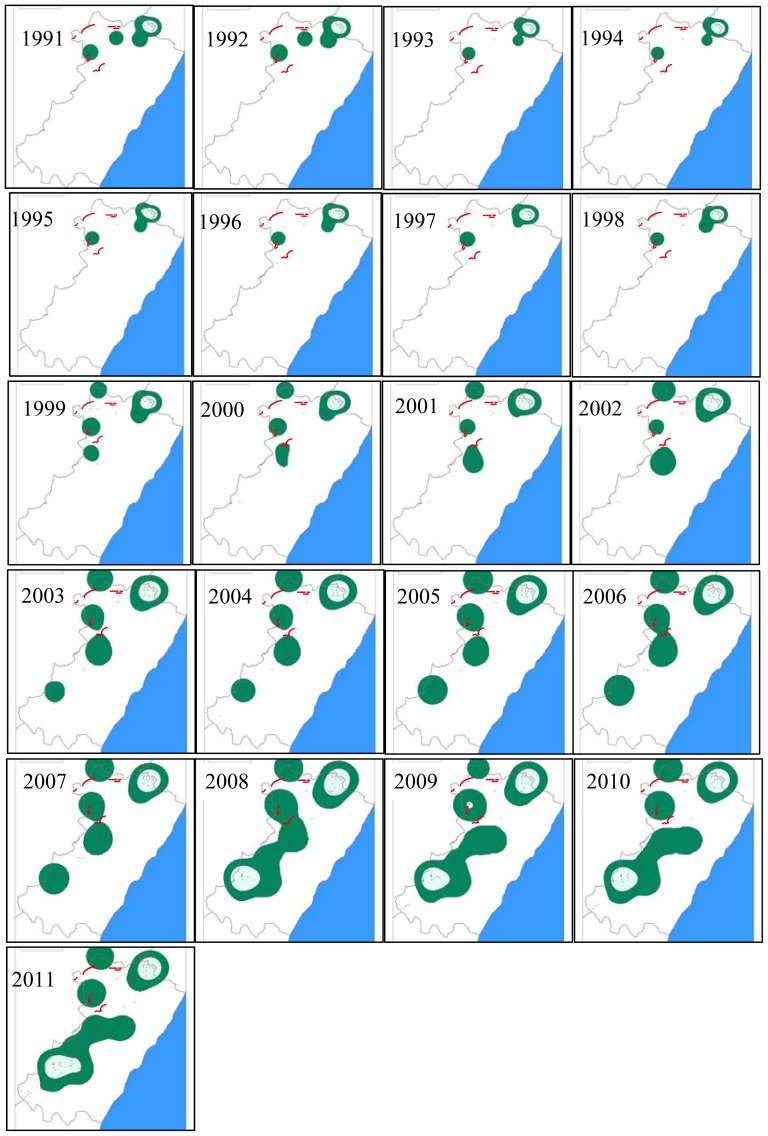
Spatial colonization of griffon vultures at the study area. Breeding range of the area occupied by griffon vultures in the study area (see details in caption of Fig. 1) by using a spatial probabilistic Kernel analysis (with 50 and 90% probabilities showed by green and shaded blue areas respectively) for the period 1991–2011 using the colonies (showed by black dots) localized in a UTM 1×1 km square. The red lines show the location of the windfarms; we note that the turbines were deployed after the breeding season of 2006. The blue part corresponds to the Mediterranean Sea.

Griffon vultures are colonial scavengers nesting on cliffs. The study population (consisting of several small colonies) occupied a surface area (calculated as a Minimum Convex Polygon) of 218 km^2^ in 1991, which expanded to 3,555 km^2^ by 2011 (see Results). The breeding population has been monitored annually since 1973, when only a few pairs remained in a single small colony, after a sharp decrease close to extinction in the 1960's due to the use of poison targeting terrestrial carnivores [25]. Nevertheless, reliable population size time-series cover 21 years (from 1991 to 2011). Exhaustive fieldwork was performed during the breeding season (from March to August) by visiting all cliffs each year and exploring (using a scope from a distance) the shelves for incubating adults or chicks. Effort was similar over the years and the probability of not detecting a nest was low owed the high visibility of the birds' faeces. A windfarm of 260 turbines was built within the study area after the 2006 breeding season (Fig. 2). The turbines causing most vulture mortality were in operation from 2007 to 2008 (during the first year 122 corpses of vultures were found below the turbines), after which the local conservation agency took actions that resulted in them being closed down [25].

Greater flamingos are waterbirds that breed in very dense colonies. The study population was established in 1992 at Punta de la Banya (Ebro Delta) and the time series for its population size covers the period from 1992 to 2011 [29]. After colonization, the flamingos occupied a small islet (0.35 ha) within the largest part of a salt-pan (3.4 km^2^), which most likely gave them good protection against terrestrial predators, since flamingos are known to be very sensitive to disturbances of any kind when breeding [24], [29]. During the first years, nests were counted once the crèche of chicks abandoned the islet and lack of disturbance was ensured. Once the colony became larger in size, nest counts were performed from aerial photographs while birds were incubating just prior to the hatching period [29]. In winter 1999, before the start of the breeding season, the owners of the salt-pan built a new dyke that reduced the surface area of the water body by 28%.

More information on monitoring methods, the study species and their local ecological features can be found elsewhere [25]–[26], [28]–[32].

### Statistical analysis

We used chronological clustering through Ward's linkage method on a Euclidean distance matrix [33] to test for the presence of breakpoints or sudden changes in population numbers following human perturbations recorded in our study [34]. With this method a set of *N* sequential clusters differing in population sizes can be identified and separated if the clusters were different enough to be considered a significant shift. Chronological clustering requires two parameters: the connectedness and the fusion level *λ*, which is a parameter quantifying clustering resolution. In our data we used a low value for the fusion level (*λ* = 0.01), which is suitable for detecting major shifts within a time series [33]. However, several larger values for parameter λ were considered to test for more subtle shifts. We expected that the years affected by the perturbations in the three time series to be detected by the chronological clustering even with low *λ* values. The groups obtained through chronological clustering can be used in a Principal Coordinate Analysis [33]. We applied a posterior test (using a conservative value of *λ* = 0.01) to assess whether the different groups detected by chronological clustering resemble each other or belong to essentially different population structures.

To analyze whether perturbations triggered the dispersal of disturbed individuals to established or new breeding areas (colonization), we calculated the minimum convex polygons (MCP) of the area occupied by the different colonies of Montagu's harriers and vultures in the study area each year and assessed whether there was a statistically significant increase in the MCP (indicating the colonization of new areas) using a chronological clustering analysis. An increase in the MCP coinciding with the perturbation would indicate an association between the environmental driver (i.e. the perturbation) and the process (i.e. dispersal) though this is only a correlation approach. We note however that a detailed monitoring of the study populations and their ecosystems each year of the study allowed us to assume that the correlative analysis was reliable. The analysis was not performed for flamingos, which occupied a single site throughout the study period.

## Results

**Figure 3:**
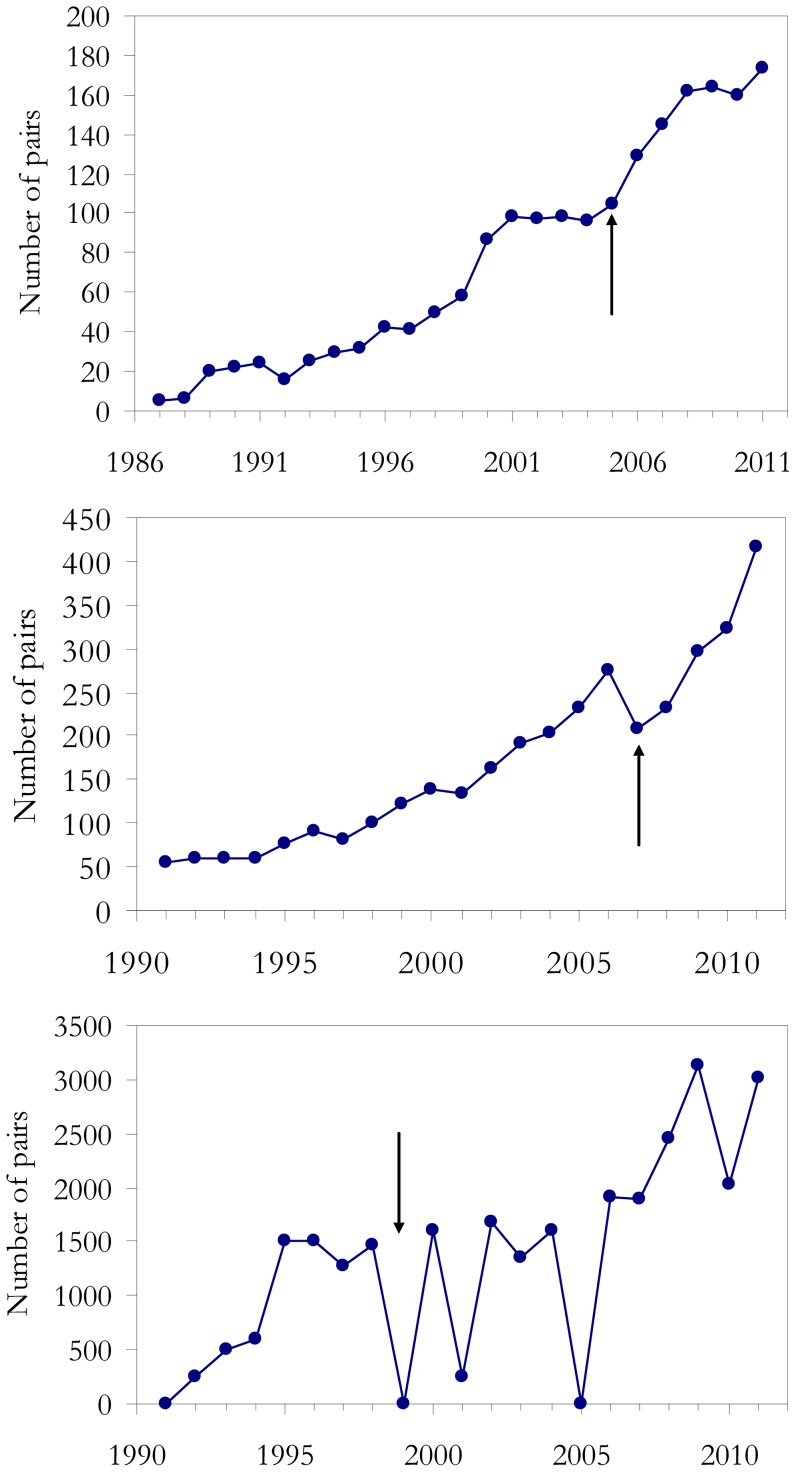
Variation in population size of the three study species over the years. Temporal changes in population numbers for the three species considered: (a) Montagu's Harrier, (b) Griffon vultures and (c) flamingos. The arrows point to the years when the anthropogenic perturbation occurred.

The populations of all three species increased steadily over the study years (Fig. 3). Strikingly, the numbers of Montagu's harriers sharply increased after the perturbation, whereas in both the griffon vulture and the greater flamingo the expected population decrease occurred. For flamingos, the consequence was the skipping of breeding of the whole colony, a phenomenon that also occurred in 2005 when there were very adverse weather conditions before the start of the reproductive season. After the population decrease, the vulture and flamingo colonies recovered quickly: in the case of vultures this was probably due to the recruitment of more birds into the breeding population [26], while the flamingos re-occupied their breeding site and slightly increased their population numbers (Fig. 3).

**Figure 4:**
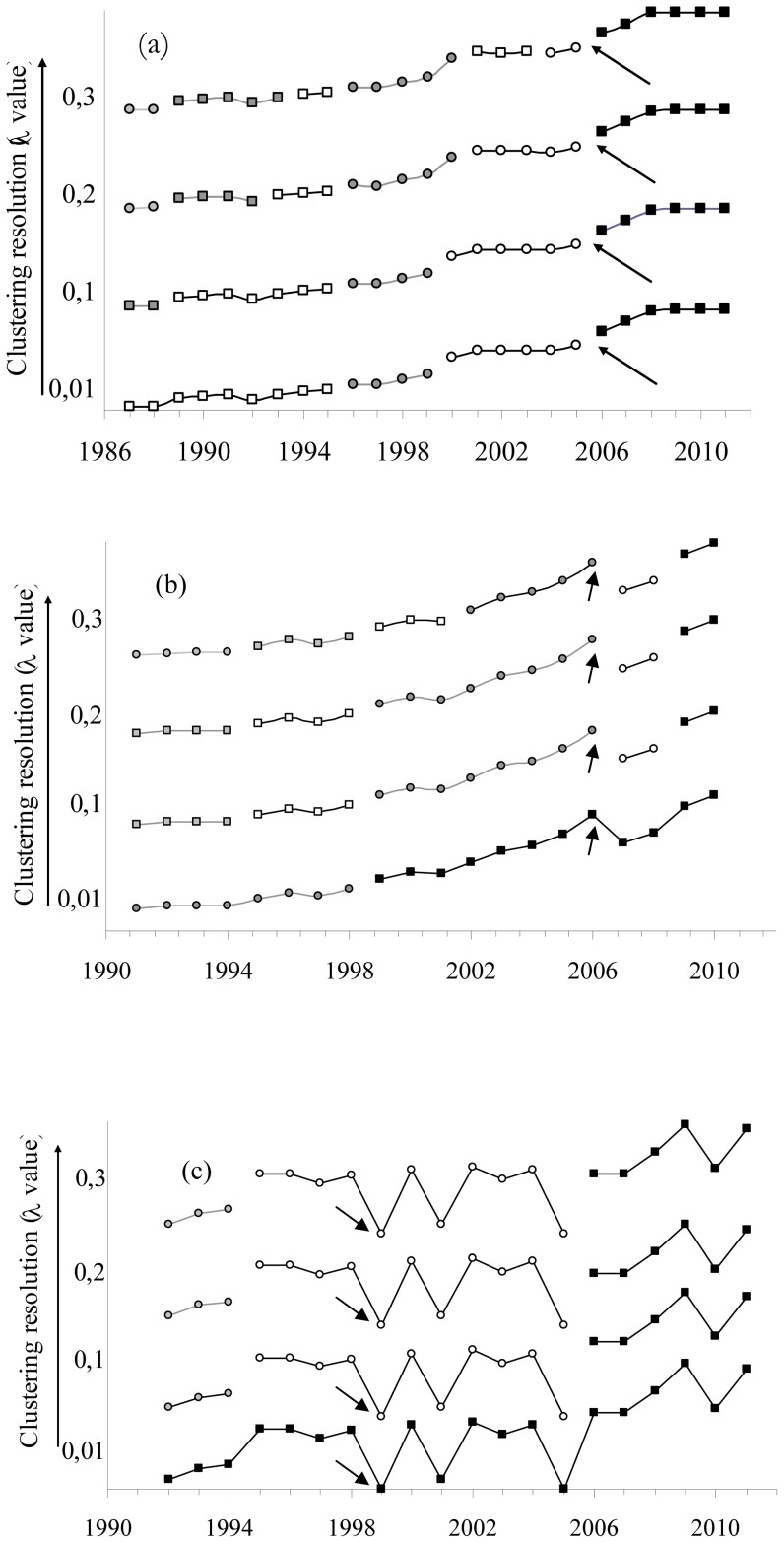
Breakpoints in the time series of the three study populations. Chronological clustering for the three study cases time series: (a) Montagu's Harrier, (b) Griffon vultures and (c) flamingos. Each panel represents a time plot of population size with statistically significant clusters denoted by different symbols and colours. For each species, four clustering time plots are shown, depending on its resolution growing with higher levels of the fusion parameter λ (Legendre & Legendre 1998). The arrows point to the years when the anthropogenic perturbation occurred in each case.

Even with small clustering resolution λ, a major shift in the size of the Montagu's harrier population was detected after the disturbance in 2006 (Fig. 4), whereas in the vultures the point of inflection in 2007 was detected only using high λ value resolutions (posterior tests for Principal Coordinate Analysis, *P*<0.001 and *P*<0.05, respectively). No statistically significant sudden change occurred for the time series of flamingos.

**Figure 5:**
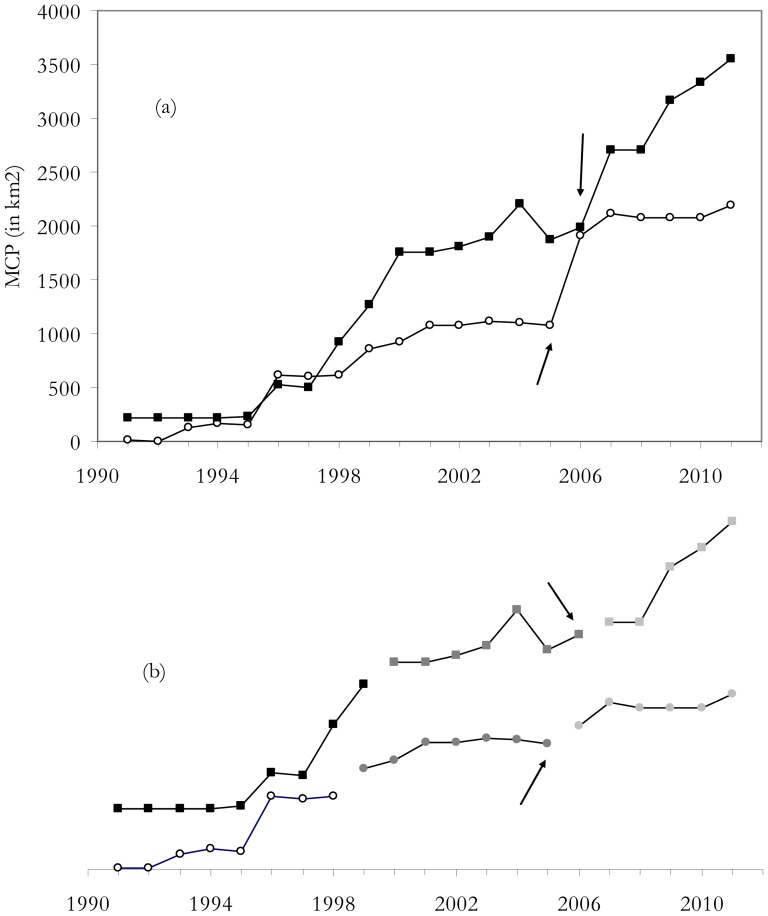
Spatial analysis of the area occupied by harriers and vultures. (a) Temporal variation of the area occupied by Montagu's harriers (white dots) and Griffon vultures (solid squares) expressed as MCP (in km2); (b) chronological clustering for harriers (dots) and vultures (squares) only for the lowest λ value resolution (0.01). The arrows show the years when the anthropogenic perturbation occurred for harriers (building of a new airport) and vultures (windfarm setup).

The spatial spread of the populations of harriers and vultures were evident since the beginning of the study (Fig. 1 and 2), corresponding to recovering population from very low numbers. Nevertheless marked larger changes were evident since 2005 for harriers and 2007 and especially 2008 for vultures coinciding with the perturbations caused by the airport and the windfarms respectively. The variability in the surface area occupied by breeding harriers and vultures in the study area (calculated as MCP) is shown in Fig. 5. MCP increased over time in the two species and reveals that, along with the increase in population size, there was also a positive trend in the colonization of new areas. Chronological clustering, using only the lowest fusion value resolution (*λ* = 0.01), shows that after the perturbations, a large shift occurred in both these species' MCP, suggesting that there was an important colonization pulse into new areas (see also Fig. 1 and 2).

## Discussion

Depending on their severity, certain perturbations can be catastrophic for some species, communities and ecosystems because habitats can be totally transformed. Thus, it is to be expected that in such cases some components of natural systems will be negatively affected, especially when perturbations are anthropogenic and add to (rather than compensate for) those of natural origin. Several theoretical studies predict that some perturbations (for instance those acting in the long-term) are liable to trigger regime shifts that lead to more impoverished states [35]. Nevertheless, predicting ecological changes in the aftermath of anthropogenic perturbations is still an important scientific challenge, because ecosystems have complex non-linear dynamics and little is known about resilience thresholds [36]–[37]. Furthermore, until relatively recently, very little research had been conducted into the short- and long-term consequences of perturbations [3], [38].

Our study shows that perturbations of human origin may cause changes in population numbers in the three study species. Nevertheless, and contrary to expectations, the building of a new airport in the core Montagu's harrier habitat strikingly coincided with a sharp increase in population numbers. The decrease in the population size of the vultures, whose adult survival and fertility were affected by the construction of a windfarm [26], was short-lived and the subsequent decrease of population size was much more subtle, probably because the turbines causing most mortality were stopped. Finally, the construction of the dyke across the salt-pan affected the quality of the breeding habitat rather than flamingo survival and, although the flamingos skipped a breeding year and re-colonized the site the following year, their population dynamics showed no change of regime. Other perturbations of natural origin (such as harsh spring temperatures before the start of the breeding season) can cause the same skipping behaviour, which is a common phenomenon in flamingos breeding in physically unstable habitats, even without the interference of any anthropogenic perturbation [39].

Recent studies have concluded that there seems to be great temporal and spatial heterogeneity in the effects of natural perturbations and that other ecological drivers acting simultaneously may also have great influence on the resilience of organisms and ecosystems [5], [38], [40]–[41]. The three study cases show that in the long term, population sizes continue to increase even despite anthropogenic perturbations, although resilience was greatest in the harriers and flamingos and slightly less in the vultures. Thus, we must ask what ecological patterns do the three study cases have in common that explain the average resilience? First, the three time series were not stationary; when we started the monitoring (in the case of flamingos, the series started with the colonization), the populations of the study species were small and living under a regime of steady population increase, meaning that suitable habitat availability was high and populations were not as yet regulated by density-dependence. This was probably the result of the important conservation legislation passed in Spain from the mid-1980s onwards that enabled the populations of many large vertebrate species to recover from low numbers [23]. Second, the three species in question are all colonial or semi-colonial birds and sociality may increase the buffering capacity of populations through ecological and behavioural processes such as colonization and positive feedback from conspecific attraction. Third, the three species are all long-lived and buffered well for decreases in fertility caused by habitat loss and deterioration; this was the case in the harriers and flamingos, but not for vultures, which suffered adult mortality [26]. The life-history traits of flamingos have evolved in ephemeral habitats such as the Etosha desert where birds have bred in only 27.5% of the last 40 years when rainfall has been high enough to ensure protection against terrestrial predators and to satisfy energy demands for breeding [42]. Species evolving in these type of habitats may be more adaptable to abrupt changes brought about by human activities (see also [12]). Finally, the three species involved are all birds with excellent dispersal abilities. Results from the MCP suggest that after the perturbation there was a subtle increase in the area occupied by harriers and vultures as a result of the colonization of new peripheral areas. Perturbations might be one of the main triggers of dispersal and can thus be responsible for the maintenance of metapopulations and gene flow within populations in many organisms. Perturbations can decrease the quality of a patch and survivors may be forced to accept the risk of dispersal and look for alternative, higher quality patches [43]–[46]. In the absence of perturbations, animals mostly remain in their patches and dispersal is precluded [47], at least, that is, until density-dependence appears or heterogeneities in habitat quality occur [48]–[50]. Although dispersal is a key trait in the dynamics of spatially discrete populations, the positive influence of perturbations in the colonization of new patches has seldom been noted [51], even though the availability of suitable empty alternative habitat is a determinant condition.

The most striking study case was that of the Montagu's harriers. The relocation of the breeding pairs affected by the habitat destruction to new areas would have maintained population size, but does not explain the sharp increase in population numbers that was detected. Analogous to the mechanism suggested by Janzen [52] to explain his ecological fitting concept, pulses of ecological disturbances may force an expansion of species' ranges that would otherwise remain in a state of geographical equilibrium. For the harriers, this should have involved not only the dispersal of established breeders, but also of the recruitment queues waiting to be incorporated into the breeding population in the core breeding area affected by the perturbation. The role of recruitment queues for buffering perturbations may play an important role in resilience in long-lived species [53]–[54].

There are numerous examples of ecosystems that have been negatively affected by anthropogenic perturbations (e.g. overfishing) whose effects, when added to those of natural perturbations (e.g. cyclones), overwhelm the components of resilience in the system [5], [55]. In our study cases and in a number of other examples [53], large anthropogenic perturbations can be buffered because resilience is sufficiently high. In some extreme cases such as the Montagu's harriers, perturbations can even benefit vulnerable species (see further examples in [56]–[57]); this is because some perturbations (natural or anthropogenic) affect the relationships between species but without necessarily damaging them (e.g. in predator-prey systems). Our results also suggest that the three study species suffered few additional anthropogenic perturbations (other than the ones documented) and occupied areas of suitable and unsaturated habitat, thereby enabling them to demonstrate their great resilience. This resilience is likely to be species-specific and, more importantly, condition-specific: the same species will have different resilience depending on the quality of the patch occupied, and even within a patch, a species' resilience will change over time. Several studies have demonstrated that even growing populations can become extinct when the frequency of perturbations is high or in the case of certain forms of mortality rate distributions caused by perturbations [58]. Thus, a major practical challenge for conservation is to predict the resilience of species, communities and ecosystems in the event of anthropogenic perturbations in a naturally variable world (see also [59]). The use of catastrophic die-offs recorded in time-series analysis shows that perturbations have profound effects on population dynamics [60]; nevertheless, this type of analysis precludes any assessment of how many populations exhibiting no sharp declines are actually resilient enough to survive perturbations. Thus, using crashes in population size time-series to identify catastrophic perturbations could be a tautology and result in a bias in the assessment of the frequency of such perturbations and, especially, of the resilience of study populations. In fact, the magnitude of a perturbation should be quantified independently of the changes in population sizes, for instance by recording the degree of changes in physical parameters and in key demographic parameters (e.g. survival, dispersal, fecundity), a task that is a further challenge in many cases. Looking for patterns and occurrence in resilience is crucial for optimizing the limited resources allocated to conservation and for predicting the future impact on ecosystems of planned anthropogenic activities.

## References

[1] Brawn JD, Robinson SK, Thompson FR III (2001) The role of disturbance in the ecology and conservation of birds. Ann Rev Ecol Syst 32: 251–276.

[2] Hsieh C, Glaser SM, Lucas AJ, Sugihara G (2005) Distinguishing random environmental fluctuations from ecological catastrophes for the North Pacific Ocean. Nature 435: 336–340. 15902256 10.1038/nature03553

[3] Lindenmayer DB, Likens GE, Franklin JF (2010) Rapid responses to facilitate ecological discoveries from major disturbances. Front Ecol Env 8: 527–532.

[4] Scheffer M, Carpenter SR (2003) Catastrophic regime shifts in ecosystems: linking theory to observation. Trends Ecol Evol 18: 648–656.

[5] Ling SD, Johnson CR, Frusher SD, Ridgway KR (2009) Overfishing reduces resilience of kelp beds to climate-driven catastrophic phase shift. Proc Nat Acad Sci USA 106: 22341–22345. 20018706 10.1073/pnas.0907529106 PMC2793314

[6] Roura-Pascual N, Hui C, Ikeda T, Leday G, Richardson DM, et al (2011) Relative roles of climatic suitability and anthropogenic influence in determining the pattern of spread in a global invader. Proc Nat Acad Sci USA 108: 220–225. 21173219 10.1073/pnas.1011723108 PMC3017164

[7] Gaillard JM, Yoccoz NG (2003) Temporal variation in survival of mammals: a case of environmental canalization? Ecology 84: 3294–3306.

[8] Blake S, Hedges S (2004) Sinking the Flagship: the Case of Forest Elephants in Asia and Africa. Cons Biol 18: 1191–1202.

[9] Massey JG (Ed.) (2007) Effects of oil on wildlife. Ninth International Effects of Oil on Wildlife Conference, Monterey, California 2007. Davis: UC Davis Wildlife Health Center.

[10] Iverson SA, Esler D (2010) Harlequin Duck population injury and recovery dynamics following the 1989 Exxon Valdez oil spill. Ecol Appl 20: 1993–2006. 21049885 10.1890/09-1398.1

[11] Matkin CO, Saulitis EL, Ellis GM, Olesiuk P, Rice SD (2008) Ongoing population-level impacts on killer whales Orcinus orca following the Exxon Valdez oil spill in Prince William Sound, Alaska. Mar Ecol Progr Ser 356: 269–281.

[12] Oro D, Tavecchia G, Genovart M (2011) Comparing demographic parameters of philopatric and immigrant individuals in a long-lived bird adapted to unstable habitats. Oecologia 165: 935–945. 20842380 10.1007/s00442-010-1773-3

[13] Darimont CT, Carlson SM, Kinnison MT, Paquet PC, Reimchen TE, et al (2009) Human predators outpace other agents of trait change in the wild. Proc Nat Acad Sci USA 106: 952–954. 19139415 10.1073/pnas.0809235106 PMC2630061

[14] Adams J, Maslin M, Thomas E (1999) Sudden climate transitions during the Quaternary. Prog Phys Geo 23: 1–36.

[15] Pickett STA, White PS (Eds) (1985) The ecology of natural disturbance and patch dynamics. San Diego: Academic Press.

[16] Solé RV, Montoya JM (2001) Complexity and fragility in ecological networks. Proc Roy Soc Lond B 268: 2039–2045. 10.1098/rspb.2001.1767 PMC1088846 11571051

[17] Thibault KM, Brown JH (2008) Impact of an extreme climatic event on community assembly. Proc Nat Acad Sci USA 105: 3410–3415. 18303115 10.1073/pnas.0712282105 PMC2265133

[18] Barnosky AD, Bell CJ, Emslie SD, Goodwin HT, Mead JI, et al (2004) Exceptional record of mid-Pleistocene vertebrates helps differentiate climatic from anthropogenic ecosystem perturbations. Proc Nat Acad Sci USA 101: 9297–9302. 15197254 10.1073/pnas.0402592101 PMC438971

[19] Sahasrabudhe S, Motter AE (2011) Rescuing ecosystems from extinction cascades through compensatory perturbations. Nature Comm 2: 170 doi:10.1038/ncomms1163. 10.1038/ncomms1163 21266969

[20] Guitart R, Sachana M, Caloni F, Croubels S, Vandenbroucke V, et al (2010) Animal poisoning in Europe. Part 3: Wildlife. Vet J 183: 260–265. 19423367 10.1016/j.tvjl.2009.03.033

[21] Wiens JA, Day RH, Murphy SM, Fraker MA (2010) Assessing Cause–Effect Relationships in Environmental Accidents: Harlequin Ducks and the Exxon Valdez Oil Spill. Curr Orn 17: 131–189.

[22] Moller AP, Mousseau TA (2011) Conservation consequences of Chernobyl and other nuclear accidents. Biol Cons 144: 2787–2798.

[23] Martínez-Abraín A, Crespo J, Jiménez J, Gómez JA, Oro D (2009) Is the historical war against wildlife over in southern Europe? Anim Cons 12: 204–208.

[24] Cezilly F, Boy V, Green RE, Hirons GJM, Johnson AR (1995) Interannual variation in greater flamingo breeding success in relation to water levels. Ecology 76: 20–26.

[25] Martínez-Abraín A, Tavecchia G, Regan H, Jiménez J, Surroca M, et al (2012) The effects of wind farms and food scarcity on a large scavenging bird species following an epidemic of bovine spongiform encephalopathy. J Appl Ecol 49: 109–117.

[26] Arroyo B, García J (2007) El aguilucho cenizo y el aguilucho pálido en España. Madrid: SEO/BirdLife.

[27] Kotze DJ, Johnson CA, O'Hara RB, Vepsäläinen K, Fowler MS (2004) Editorial: The Journal of Negative Results in Ecology and Evolutionary Biology. J Neg Res 1: 1–5.

[28] Limiñana R, Surroca M, Miralles S, Urios V, Jiménez J (2006) Population trend and breeding biology of Montagu's Harrier *Circus pygargus* in a natural vegetation site in northeast Spain. Bird Stud 53: 126–131.

[29] Curcó A, Vidal F, Piccardo J (2009) Conservation and management of the Greater Flamingo *Phoenicopterus roseus* . Flamingo 1: 37–43.

[30] Limiñana R, García J, González J, Guerrero Á, Lavedán J, et al (2012) Philopatry and natal dispersal of Montagu's harriers (*Circus pygargus*) breeding in Spain: a review of existing data. Eur J Wild Res 58 (in press).

[31] Soutullo A, Limiñana R, Urios V, Surroca M, Gill JA (2006) Density-dependent regulation of population size in colonial breeders: Allee and buffer effects in the migratory Montagu's harrier. Oecologia 149: 543–552. 16794831 10.1007/s00442-006-0465-5

[32] Genovart M, Surroca M, Martínez-Abraín A, Jiménez J (2008) Parity in Fledging Sex Ratios in a Dimorphic Raptor, Montagu's Harrier *Circus pygargus* . Zool Stu 47: 11–16.

[33] Zuur AF, Ieno EN, Smith GM (2007) Analysing Ecological Data. New York: Springer.

[34] Andersen T, Carstensen J, Hernández-García E, Duarte CM (2009) Ecological thresholds and regime shifts: approaches to identification. Trends Ecol Evol 24: 49–57. 18952317 10.1016/j.tree.2008.07.014

[35] Solé RV (2007) Scaling laws in the drier. Nature 449: 151–153. 17851503 10.1038/449151a

[36] Jackson ST, Betancourt JL, Booth RK, Gray ST (2009) Ecology and the ratchet of events: climate variability, niche dimensions, and species distributions. Proc Nat Acad Sci 106: 19685–19692. 19805104 10.1073/pnas.0901644106 PMC2780932

[37] Thrush SF, Hewitt JE, Dayton PK, Coco G, Lohrer AM, et al (2009) Forecasting the limits of resilience: integrating empirical research with theory. Proc Roy Soc Lond B 276: 3209–3217. 10.1098/rspb.2009.0661 PMC2817168 19553254

[38] Romme WH, Boyce MS, Gresswell R, Merrill EH, Minshall GW, et al (2011) Twenty Years after the 1988 Yellowstone Fires: Lessons about Disturbance and Ecosystems. Ecosystems 14: 1196–1215.

[39] Balkiz Ö, Béchet A, Rouan L, Choquet R, Germain C, et al (2010) Experience-dependent natal philopatry of breeding Greater Flamingos. J Anim Ecol 79: 1045–1056. 20626499 10.1111/j.1365-2656.2010.01721.x

[40] Avise JC, Hubbell SP, Ayala FJ (2008) In the Light of Evolution, Volume II: Biodiversity and Extinction. Washington: National Academy of Sciences. 25009919

[41] Fernández-Chacón A, Bertolero A, Amengual A, Tavecchia G, Homar V, et al (2011) Spatial heterogeneity in the effects of climate change on the population dynamics of a Mediterranean tortoise. Glob Change Biol 17: 3075–3088.

[42] Johnson AR, Cézilly F (2007) The Greater Flamingo. London: T. & A. D. Poyser.

[43] Oro D, Pradel R, Lebreton JD (1999) Food availability and nest predation influence life history traits in Audouin's gull, *Larus audouinii* . Oecologia 118: 438–445. 28307411 10.1007/s004420050746

[44] Davidson Z, Valeix M, Loveridge AJ, Madzikanda H, Macdonald DW (2011) Socio-spatial behaviour of an African lion population following perturbation by sport hunting. Biological Conservation 144: 114–121.

[45] McDowall R (2010) Why be amphidromous: expatrial dispersal and the place of source and sink population dynamics? Reviews in Fish Biology and Fisheries 20: 87–100.

[46] Lachish S, Miller KJ, Storfer A, Goldizen AW, Jones ME (2011) Evidence that disease-induced population decline changes genetic structure and alters dispersal patterns in the Tasmanian devil. Heredity 106: 172–182. 20216571 10.1038/hdy.2010.17 PMC3183847

[47] Parsons DM, Morrison MA, Slater MJ (2010) Responses to marine reserves: Decreased dispersion of the sparid *Pagrus auratus* . Biol Cons 143: 2039–2048.

[48] Poethke HJ, Hovestadt T (2002) Evolution of density- and patch-dependent dispersal rates. Proc Roy Soc Lond B 269: 637–645. 10.1098/rspb.2001.1936 PMC1690934 11916481

[49] Oro D, Cam E, Pradel R, Martínez-Abrain A (2004) Influence of food availability on demography and local population dynamics in a long-lived seabird. Proc Roy Soc Lond B 271: 387–396. 10.1098/rspb.2003.2609 PMC1691609 15101698

[50] Gaillard JM, Hewison AJM, Kjellander P, Pettorelli N, Bonenfant C, et al (2008) Population density and sex do not influence fine-scale natal dispersal in roe deer. Proc Roy Soc B Biol Sci 275: 2025–2030. 10.1098/rspb.2008.0393 PMC2596358 18505718

[51] Akçakaya HR, Baur B (1996) Effects of population subdivision and catastrophes on the persistence of a land snail metapopulation. Oecologia 105: 475–483. 28307140 10.1007/BF00330010

[52] Janzen DH (1985) On Ecological Fitting. Oikos 45: 308–310.

[53] Votier S, Birkhead TR, Oro D, Trinder M, Grantham MJ, et al (2008) Recruitment and survival of immature seabirds in relation to oil spills and climate variability. J Anim Ecol 77: 974–983. 18624739 10.1111/j.1365-2656.2008.01421.x

[54] Penteriani V, Ferrer M, Delgado MM (2011) Floater strategies and dynamics in birds, and their importance in conservation biology: towards an understanding of nonbreeders in avian populations. Anim Cons 14: 233–241.

[55] Adger WN, Hughes TP, Folke C, Carpenter SR, Rockström J (2005) Social-Ecological Resilience to Coastal Disasters. Science 309: 1036–1039. 16099974 10.1126/science.1112122

[56] Leighton PA, Horrocks JA, Kramer DL (2010) Conservation and the scarecrow effect: Can human activity benefit threatened species by displacing predators? Biol Cons 143: 2156–2163.

[57] Muhly TB, Semeniuk C, Massolo A, Hickman L, Musiani M (2011) Human Activity Helps Prey Win the Predator-Prey Space Race. PLoS ONE 6: e17050. 21399682 10.1371/journal.pone.0017050 PMC3047538

[58] Mangel M, Tier C (1994) Four Facts Every Conservation Biologists Should Know about Persistence. Ecology 75: 607–614.

[59] Hill D, Arnold R (2012) Building the evidence base for ecological impact assessment and mitigation. J Appl Ecol 49: 6–9.

[60] Reed DH, O'Grady JJ, Ballou JD, Frankham R (2003) The frequency and severity of catastrophic die-offs in vertebrates. Anim Cons 6: 109–114.

